# Prevalence and factors associated with laboratory-confirmed cases of select enteric infections in three Ethiopian communities, 2018–2022

**DOI:** 10.1371/journal.pgph.0005021

**Published:** 2025-08-11

**Authors:** Devin LaPolt, Binyam Moges Azmeraye, Zhanpeng Kuang, Desalegne Degefaw, Amete Mihret Teshale, Silvia Alonso, Yadeta Dessie, Melanie Lopez, Alem Abrha Kalayu, Gashaw Andargie, Barbara Kowalcyk

**Affiliations:** 1 Department of Food Science and Technology, The Ohio State University, Columbus, Ohio, United States of America; 2 The Ohio State University Global One Health Initiative, Eastern Africa Regional Office, Addis Ababa, Ethiopia; 3 Division of Biostatistics, College of Public Health, The Ohio State University, Columbus, Ohio, United States of America; 4 National Clinical Bacteriology and Mycology Reference Laboratory, Ethiopian Public Health Institute, Addis Ababa, Ethiopia; 5 International Livestock Research Institute, Addis Ababa, Ethiopia; 6 School of Public Health, College of Health and Medical Sciences, Haramaya University, Harar, Ethiopia; 7 Veterinary Preventive Medicine, College of Public Health, The Ohio State University, Columbus, Ohio, United States of America; 8 Department of Microbiology, Immunology and Parasitology, School of Medicine; College of Health Sciences, Addis Ababa University, Addis Ababa, Ethiopia; 9 Institute of Public Health, College of Medicine and Health Sciences, University of Gondar, Gondar, Ethiopia; 10 Translational Data Analytics Institute, The Ohio State University, Columbus, Ohio, United States of America; 11 Milken School of Public Health, The George Washington University, Washington D.C., United States of America; PLOS: Public Library of Science, UNITED STATES OF AMERICA

## Abstract

Enteric diseases are major contributors to morbidity and mortality worldwide; however, in Ethiopia, information on the prevalence of enteric infections and associated trends is limited. Understanding the epidemiology of enteric infections is necessary for determining disease burden and allocating resources. The objective of this study was to estimate the prevalence of laboratory-confirmed infections associated with select parasitic and bacterial pathogens in three Ethiopian hospitals, assess trends, and identify associated factors. Laboratory and patient metadata for stool samples tested at clinical laboratories in Addis Ababa, Gondar, and Harar in Ethiopia from 2018 through 2022 were collected and digitized. Descriptive statistics were used to summarize laboratory results and assess trends in sample submission and infection. Prevalence of laboratory-confirmed infection and 95% confidence intervals were estimated by pathogen using binomial proportion testing and logistic regression. Univariate and multivariable logistic regression were used to identify associated factors. A total of 48,643 samples were included in the analysis. Prevalence estimates for parasitic infection were 5.23% [95%CI:4.87%,5.62%], 17.48% [95%CI:17.04%,17.93%], and 15.69% [95%CI:14.57%,16.85%] in Addis Ababa, Gondar, and Harar, respectively. Prevalence estimates for bacterial infection were 0.25% [95%CI:0.07%,0.65%] and 7.59% [95%CI:5.97%,9.50%] in Addis Ababa and Gondar, respectively; stool samples were not tested for bacterial pathogens in Harar. Stool sample submission and enteric infection detection varied by year at each site. Age, season, and year of submission were identified as factors associated with the detection of enteric pathogens in stool samples. Prevalence estimates differed across study sites and testing was not conducted for many enteric pathogens associated with diarrhea. Additional research to understand the scope of enteric infection is necessary for resource allocation toward robust diagnostic procedures and increased laboratory capacity for stool testing. Efforts to mitigate enteric infection should utilize seasonal and geographic infection trends to anticipate areas in need of additional resources.

## Introduction

Globally, enteric infections and diarrheal diseases cause over one million deaths yearly, with African countries bearing a disproportionate amount of this burden [1–3]. For example, the annual incidence of diarrheal disease from bacterial and parasitic pathogens is over 20% higher in the African region compared to global estimates [4]. The incidence of parasitic and enteric bacterial infections is often underestimated due to factors like low healthcare-seeking behavior and suboptimal surveillance.

Globally, enteric parasitic infections cause an estimated 400 million illnesses annually, with Africa being disproportionately affected, with incidence rates nearly twice the global estimates [5]. Parasites like *Giardia lamblia*, *Entamoeba histolytica*, and *Ascaris lumbricoides* are associated with diarrheal disease, particularly in children. Social, environmental, and geographic factors such as dietary patterns, use of unimproved drinking water or sanitation, and rainfall can influence the prevalence of infection [1,6,7]. In Ethiopia, a multi-region study of school children estimated the pooled prevalence of parasitic infection to be 46.09% [8]; however, little is known about the scope of parasitic infection in adult populations. Parasitic infections, such as Giardiasis, are thought to be largely underreported due to limited surveillance and inadequate healthcare-seeking behaviors, making estimating the burden of parasitic diarrheal disease challenging [9–11].

Bacterial pathogens, such as non-typhoidal *Salmonella* (NTS), *Shigella* spp., *Campylobacter* spp., Shiga toxin-producing *Escherichia coli* (STEC), are also major causes of diarrhea globally, with water and waste management infrastructure, and environmental and lifestyle/social factors greatly influencing diarrheal prevalence [2,12,13]. In Africa, the median incidence rates associated with NTS and *Shigella* are thought to be much lower than global estimates, despite rates of diarrheal disease being much higher [4]. However, there is significant uncertainty in these estimates due to a lack of surveillance data in many African countries. Additionally, routine methods for bacterial detection often underestimate the prevalence [14,15]. In Ethiopia, surveillance studies have found the prevalence of enteric bacteria such as *Shigella* spp, *Salmonella* spp, and enterohemorrhagic *Escherichia coli* (EHEC) to range from 13 – 25% in children under the age of five [16,17] and 0.8% - 13.2% in healthy adult food handlers [18–20]. Additionally, a study of adults with HIV in northeast Ethiopia estimated the prevalence of *Salmonella* to be 5.4% [21]. However, estimates of the prevalence of bacterial infection in the general adult population and children over the age of five are unknown.

Limitations in surveillance and reporting suggest that existing estimates may underestimate the burden of enteric infection and diarrheal disease in Ethiopia [22–24]. For example, the Strengthening Laboratory Management Toward Accreditation (SLMTA) program found that limited awareness, lack of quality manuals, and discordance with other laboratory activities were common challenges in Ethiopian laboratories [25]. Additionally, the Ethiopian Public Health Institute has identified strengthening quality assurance, implementation of robust quality management systems, and enhancing referral and testing capacity as major capacity-building objectives [26]. These activities, coupled with centralized healthcare data management, could allow for a better understanding of the scope of enteric infection in Ethiopia. This, in turn, can allow for improved burden of disease estimates, informed allocation of hospital resources, and robust diagnosis and treatment for enteric infection to ultimately improve infectious disease management practices in Ethiopia.

Laboratory capacity can also limit surveillance and reporting, especially in low- and middle-income countries (LMICs) where infrastructure, improved sanitation, and access to healthcare and treatment are frequently inconsistent or in short supply [27–30]. Moreover, diagnostic facilities, specialized medical personnel, treatment for enteric infection or diarrheal disease, and data management infrastructure are not centralized, and capacity is often limited [31,32]. A country’s diagnostic capacity can restrict treatment as traditional diagnostic procedures are often not robust (i.e., not testing for all sources of infection, not using molecular methods), resulting in ineffective treatment [15]. In Ethiopia, current burden estimates are uncertain as most research is focused on specific population groups, such as children under five [33–35]. This challenge is not unique to Ethiopia, as many LMICs focus diarrheal disease research on certain population groups due to a lack of resource availability.

Limited information on the scope of enteric infection has resulted in gaps in understanding of the burden from enteric pathogens and subsequent diarrheal disease in Ethiopia, making resource allocation and infectious disease control challenging. For example, routine national surveillance and mandatory reporting is only conducted for 20 diseases on a daily or weekly basis in Ethiopia, including three (cholera, dysentery, typhoid fever) that are commonly transmitted through food or water and associated with diarrhea [36]. Additionally, many important enteric pathogens associated with foodborne and waterborne transmission (e.g., NTS, *Campylobacter* spp., STEC) are not included in routine surveillance efforts. Estimating the prevalence of enteric infections can lead to a better understanding of diarrheal disease and factors influencing the risk of illness, and further inform resource allocation. The objective of this study was to retrospectively estimate the prevalence of parasitic and bacterial infections at three hospitals in Ethiopia from laboratory and hospital records, assess trends in stool sample submission, and identify factors linked with enteric infection.

## Methods

A secondary analysis of health facility-based surveillance data from patients submitting stool samples to three Ethiopian hospitals between January 2018 and December 2022 was conducted. Parasitology and microbiology records and registration logbooks from various departments (i.e., Inpatient Department, Outpatient Department, Emergency Department, Pediatric Department) were obtained from Yekatit 12 Hospital (Addis Ababa), University of Gondar Hospital (Gondar), and Hiwot Fana Comprehensive Specialized Hospital (Harar). Records were transcribed by trained data collectors into an electronic database (Microsoft Access). Data fields included study site (hospital), patient age (years), patient sex, date of stool collection, date of record entry, laboratory type (parasitology or microbiology), and laboratory results (parasitic and bacterial). Periodic data checks were conducted to ensure data quality. Dates reported in the Ethiopian calendar were converted to the Gregorian calendar. Patients with incomplete clinical data (missing information on age, sex, date of stool collection, laboratory test results) were excluded from the analysis. Outlier values that were suspected to be data entry errors (e.g., age above 100 years) were also excluded from analysis. Additionally, stool samples positive for non-enteric pathogens and stool samples with undetermined or unclear laboratory diagnoses were excluded from analysis.

Methods for processing and testing stool samples varied by site. In Addis Ababa, four departments (Emergency Adult Outpatient Department (OPD), Adult OPD, Emergency Pediatrics OPD, Inpatient) ordered patient stool samples. Stool samples were submitted to either the central parasitology or central microbiology laboratory based on physician orders and tested for parasitic pathogens (central parasitology laboratory) or *Salmonella* and *Shigella* (central microbiology laboratory). Samples were also screened for *Vibrio cholerae* during periods of suspected outbreak. In Gondar, five departments (Adult Emergency OPD, Adult OPD, Pediatrics Emergency OPD, Pediatrics OPD, Inpatient) ordered patient stool samples. Stool samples were submitted to the central laboratory for testing based on physician orders and tested for parasitic pathogens (central parasitology laboratory) or *Salmonella* and *Shigella* (central microbiology laboratory). Samples were also screened for *Vibrio cholerae* during periods of suspected outbreak. In Harar, three departments (Emergency OPD, Central Intensive Care Unit (ICU), Inpatient) ordered patient stool samples, which were submitted to a central parasitology laboratory and tested for parasites. No bacterial testing was performed as there was no microbiology laboratory during the study period. Stool samples at all hospitals were tested using ISO protocol 15189 [37]; all parasitology testing was conducted using wet mount microscopy, and bacterial testing was conducted using traditional culturing and microscopy.

Descriptive statistics were used to summarize socio-demographic characteristics and laboratory results. The prevalence of laboratory-confirmed enteric infection with 95% Clopper-Pearson (Exact) confidence intervals was estimated by pathogen using logistic regression and binomial proportion testing. Additionally, for this study, soil-transmitted helminths (STH) included *Ascaris lumbricoides*, Hookworm (*Ancylostoma duodenale*, *Necator americanus*), *Strongyloides stercoralis*, and *Trichuris trichiura* [38,39]. Since stool sample processing varied between the three study sites, data were assessed separately by study site, and site-specific models were developed. Trends in codetections (i.e., single stool samples reporting detection of more than one enteric pathogen) were assessed using correlation analysis. Seasonal trends in stool sample submission were assessed by site using Poisson regression. Univariate and multivariable models were constructed, and interaction effects were assessed.

Univariable binary logistic regression was used to identify potential factors for enteric infection. Site-specific multivariable binary logistic regression models were constructed, and factors were included in multivariable models based on significance in univariate models (cut point 0.2). Effect modification was assessed by comparing adjusted estimates to crude estimates, and interaction terms were included based on significance; model goodness-of-fit was assessed using the Hosmer-Lemeshow Goodness-of-fit test [40]; and diagnostic statistics were used to assess the influence of individual observations on the model. Pairwise comparisons for categorical variables were assessed with a Bonferroni adjustment; estimates were similar, and unadjusted estimates were reported. A codebook can be found in the S1 File.

Microsoft Access was used for data entry, and all statistical analyses were performed using SAS 9.4 for Windows (SAS Institute Inc., USA). Ethical approvals were obtained from all participating institutions including The Ohio State University [2020H0347], The George Washington University [NCR235242], Yekatit 12 Hospital [Protocol 68/20], University of Gondar Hospital [V/P/RCS/05/624/2020], and Haramaya University Institutional Ethical Review Committee [IHRERC/021/2021, IHRERC/036/2023]. Consent was not obtained from study participants since this was a retrospective analysis of existing medical records. Data was accessed 1/6/2021 – 31/10/2021 and 1/4/2023 – 30/6/2023 at all three sites by the study team. The authors did not have access to information that could identify individual participants during or after data collection.

## Results

Demographic and laboratory result data were obtained from a total of 52,913 stool samples across the three sites during the study period. Of these, 48,643 (91.93%) were included in the analysis (Table 1). The number of stool samples from males and females was evenly distributed at each site, with there being a slightly higher proportion of females compared to males in Gondar. The mean age was similar across sites, with the age distribution at Addis Ababa being skewed towards the pediatric population. The study included patients with ages ranging from 0 to 100 years.

**Table 1:** Patient demographics by study site, 2018–2022.

		All Patients	Addis Ababa	Gondar	Harar
Laboratory Records	Total N (%)	52,913 (100%)	19,043 (35.99%)	29,620 (55.98%)	4,250 (8.03%)
	Included N (%)^a^	48,643 (91.93%)	15,431 (81.03%)	29,215 (98.63%)	3,997 (94.05%)
Age(years)	Mean (Std Dev)	26.02 (18.62)	23.07 (20.81)	27.66 (17.26)	25.38 (17.73)
	Median (IQR)	25.00 (24.00)	20.00 (31.00)	26.00 (16.00)	24.00 (23.00)
Sex	Male	21,896 (45.01%)	7,373 (47.78%)	12,475 (42.70%)	2,048 (51.24%)
	Female	26,747 (54.99%)	8,058 (52.22%)	16,740 (57.30%)	1,949 (48.76%)

^a^4,270 samples (Addis Ababa = 3,612, Gondar = 405, Harar = 253) were excluded from analyses due to missing age, sex, date of collection, lab results, or improbable values.

**Fig 1:**
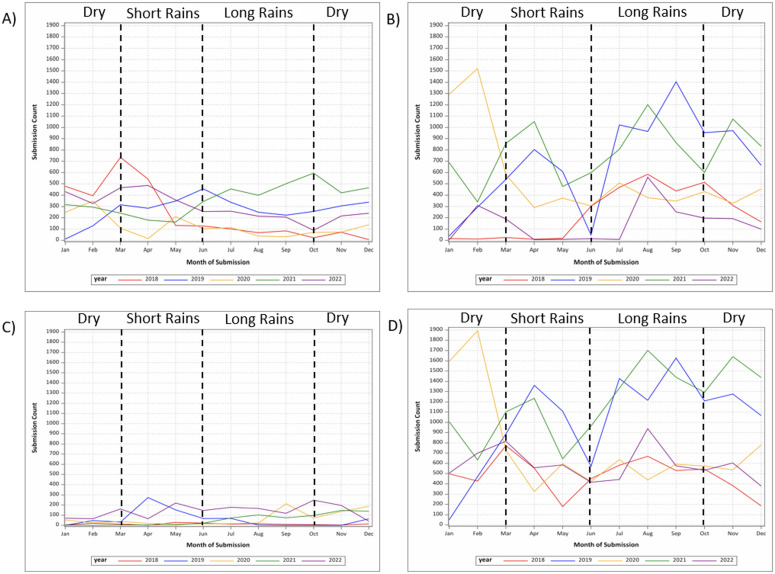
Time trends in stool sample submission across study sites in Ethiopia, 2018–2022. A) Addis Ababa; B) Gondar C) Harar D) All sites.

Stool sample submission trends varied by site (Fig 1). At all three sites, multivariable Poisson regression models of season (p-value: < 0.0001) and year of submission (p-value: < 0.0001) identified significant variation in stool sample submission across years. Stool sample submissions were lower in 2020 compared to 2018 and 2019 in Addis Ababa. In Gondar, stool sample submissions were lower in 2018 and 2022 compared to other years. In Harar, stool sample submissions were lower in 2018, 2020, and 2021 compared to other years. Additionally, stool sample submission trends also varied by season at each site. In Addis Ababa, stool sample submissions were generally lower during the short (March - May) and long rains (June - September) seasons compared to the dry season (October - February), while stool sample submissions were generally lower during the short rains season in Gondar and Harar.

**Fig 2:**
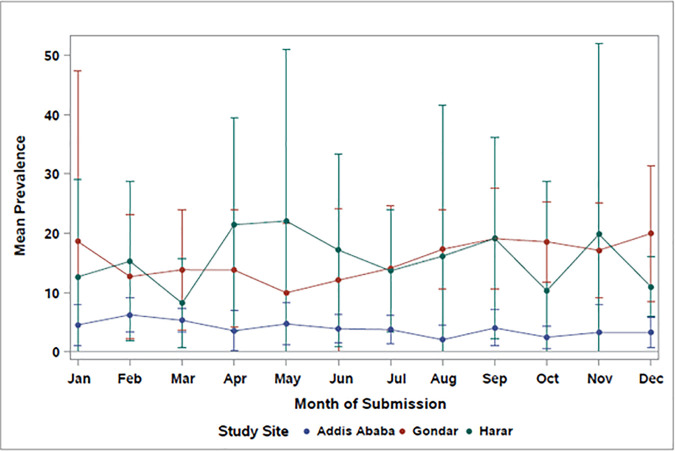
Mean monthly prevalence of enteric infection in Ethiopia by study site, 2018–2022.

Of the stool samples submitted, 46,143 (94.86%) and 2,500 (5.14%) were analyzed for parasites and bacterial pathogens, respectively. The overall parasite prevalence estimates were 5.23% (95% CI: 4.87%, 5.62%), 17.48% (95% CI: 17.04%, 17.93%), and 15.69% (95% CI: 14.57%, 16.85%) in Addis Ababa, Gondar, and Harar, respectively (Table 2 and Fig 2). The most prevalent parasites detected in Addis Ababa were *Entamoeba histolytica* [3.70% (95% CI: 3.39%, 4.02%)] and *Giardia lamblia* [1.06% (95% CI: 0.90%, 1.25%)]. The most prevalent parasites in Gondar were *Entamoeba histolytica* [9.70% (95% CI: 9.36%, 10.05%)], soil-transmitted helminths (STH) [4.05% (95% CI: 3.83%, 4.29%)], and *Giardia lamblia* [3.42% (95% CI: 3.22%, 3.64%)]. The most prevalent parasites in Harar were *Giardia lamblia* [7.31% (95% CI: 6.52%, 8.16%)] and *Entamoeba histolytica* [7.28% (95% CI: 6.49%, 8.13%)]. The prevalence of *Giardia lamblia* was much higher in Harar compared to Addis Ababa and Gondar while the prevalence of STH in Gondar was substantially higher than the prevalence of STH in the other two study sites.

**Table 2:** Prevalence of enteric pathogens by study site in Addis Ababa, Gondar, and Harar, Ethiopia, 2018–2022.

Pathogen	Prevalence^a^ [%, (95% Exact Confidence Interval)]			
	Combined	Addis Ababa	Gondar	Harar
*Entamoeba histolytica*	7.69 (7.45, 7.94)	3.70 (3.39, 4.02)	9.70 (9.36, 10.05)	7.28 (6.49, 8.13)
*Enterobius vermicularis*	0.15 (0.12, 0.19)	0.05 (0.02, 0.10)	0.20 (0.15, 0.26)	0.20 (0.09, 0.39)
*Fasciola hepatica*	0.00 (0.00, 0.01)	0^b^ (-, -)	0.00 (0.00, 0.02)	0 (-, -)
*Giardia lamblia*	3.05 (2.90, 3.21)	1.06 (0.90, 1.25)	3.42 (3.22, 3.64)	7.31 (6.52, 8.16)
*Hymenolepis nana*	0.26 (0.22, 0.31)	0.12 (0.07, 0.20)	0.30 (0.24, 0.37)	0.48 (0.29, 0.74)
*Hymenolepis* spp	0.00 (0.00, 0.02)	0.01 (0.00, 0.04)	0.00 (0.00, 0.02)	0^b^ (-, -)
*Schistosoma mansoni*	0.67 (0.60, 0.75)	0.01 (0.00, 0.04)	1.07 (0.95, 1.20)	0.13 (0.04, 0.29)
Soil-transmitted Helminths (STH^c^)	2.58 (2.44, 2.73)	0.22 (0.15, 0.31)	4.05 (3.83, 4.29)	0.33 (0.17, 0.56)
*Ascaris lumbricoides*	1.33 (1.23, 1.44)	0.05 (0.02, 0.10)	2.12 (1.96, 2.30)	0.13 (0.04, 0.29)
*Ascaris* spp	0.03 (0.02, 0.06)	0.06 (0.02, 0.11)	0.02 (0.01, 0.05)	0.05 (0.01, 0.18)
Hookworm (*Ancylostoma duodenale*, *Necator americanus*)	1.07 (0.98, 1.17)	0.05 (0.02, 0.10)	1.71 (1.56, 1.86)	0.08 (0.02, 0.22)
*Strongyloides stercoralis*	0.18 (0.14, 0.22)	0.03 (0.01, 0.07)	0.27 (0.21, 0.34)	0.08 (0.02, 0.22)
*Strongyloides* spp	0.02 (0.01, 0.04)	0.02 (0.00, 0.06)	0.02 (0.01, 0.05)	0^b^ (-, -)
Whipworm (*Trichuris trichiura*)	0.02 (0.01, 0.03)	0.01 (0.00, 0.04)	0.02 (0.01, 0.05)	0^b^ (-, -)
*Taenia* spp	0.23 (0.19, 0.28)	0.12 (0.07, 0.20)	0.31 (0.25, 0.38)	0.08 (0.02, 0.22)
**Overall parasite**	13.65 (13.34, 13.97)	5.23 (4.87, 5.62)	17.48 (17.04, 17.93)	15.69 (14.57, 16.85)
*Salmonella* spp	0.08 (0.01, 0.29)	0.13 (0.02, 0.46)	0^b^ (-, -)	NA^d^
*Shigella* spp	2.92 (2.30, 3.66)	0.19 (0.04, 0.55)	7.59 (5.97, 9.50)	NA
**Overall bacteria** ^e^	2.96 (2.33, 3.70)	0.25 (0.07, 0.65)	7.59 (5.97, 9.50)	NA

^a^ Some samples counted more than once due to codetections.

^b^ Zero prevalence may be due to a lack of testing.

^c^ Includes *Ascaris lumbricoides*, Hookworm, *Trichuris trichiura*, *Strongyloides stercoralis*.

^d^ Not Applicable; no samples tested for bacterial pathogens during the study period (no microbiology laboratory available).

^e^ Samples were only tested for *Salmonella* and *Shigella*.

Of 2,500 samples tested for bacterial pathogens in two sites, the overall prevalence estimates were 0.25% (95% CI: 0.07%, 0.65%) and 7.59% (95% CI: 5.97%, 9.50%) for Addis Ababa and Gondar, respectively (Table 2 and S2 File). In Addis Ababa, *Shigella* [0.19% (95% CI: 0.04%, 0.55%)] and *Salmonella* [0.13% (95% CI: 0.02%, 0.46%)] were the most prevalent bacterial pathogens, and *Shigella* [7.59% (95% CI: 5.97%, 9.50%)] was the only bacterial pathogen detected in Gondar. No bacterial testing was conducted on samples at Harar during the study period.

**Fig 3:**
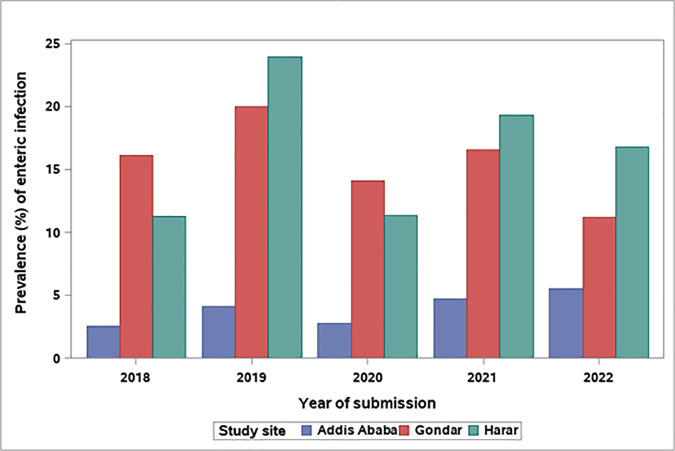
Annual prevalence of enteric infection from all tested pathogens by study site in Ethiopia, 2018–2022.

The overall prevalence of parasitic infection in Harar increased seasonally with a sharp increase in the short rains season (March - May) and variable increases and decreases throughout the long rains season (June - September). The mean prevalence of enteric infection remained fairly consistent by month in Addis Ababa with slight increases in February and March. In Gondar, mean prevalence estimates of enteric infection from bacterial and parasitic pathogens were variable across the year. The prevalence of enteric infection also varied by year across study sites. In Addis Ababa, prevalence estimates remained generally consistent. However, in Gondar and Harar there was significant variability in prevalence estimates by year (Fig 3).

Of 6,299 samples positive for at least one parasitic pathogen, two or more pathogens were detected in 457 (7.26%) stool samples. Pathogens frequently co-detected in samples included *Entamoeba histolytica*, *Giardia lamblia*, and STH, with the number of co-detected pathogens ranging from 2 – 4 pathogens. Of the 74 samples that tested positive for at least one bacterial pathogen, two or more pathogens were only detected in one sample. Correlation in codetections varied by study site. In Addis Ababa, there was a positive correlation between *Ascaris lumbricoides* and *Hymenolepis nana*, as well as *Enterobius vermicularis* and Hookworm. In Gondar, there was a positive correlation between *Ascaris lumbricoides* and *Taenia* spp, as well as *Entamoeba histolytica* and *Schistosoma mansoni*. In Harar, there was a slight negative correlation between *Entamoeba histolytica* and *Giardia lamblia*. Additional codetection data are presented in S3 File.

**Fig 4:**
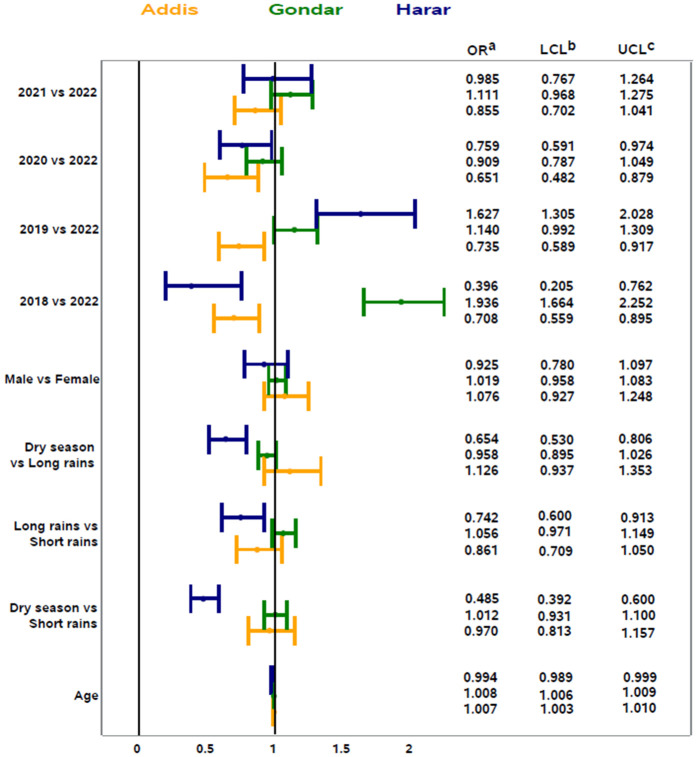
Univariate logistic regression of factors associated with enteric infection by study site in Ethiopia, 2018–2022. ^a^Unadjusted odds ratio, ^b^Wald 95% lower confidence limit, ^c^Wald 95% upper confidence limit.

In univariate logistic regression models, age and year of stool sample submission were significantly associated with enteric infection in Addis Ababa and Gondar. In Harar, age, season, and year of submission were significantly associated with the detection of enteric infection. Additionally, the direction of association varied by site for age and year of submission (Fig 4). In Addis Ababa and Gondar, the unadjusted odds of enteric infection increased slightly with age, while in Harar, the unadjusted odds of enteric infection decreased slightly with increasing age.

**Fig 5:**
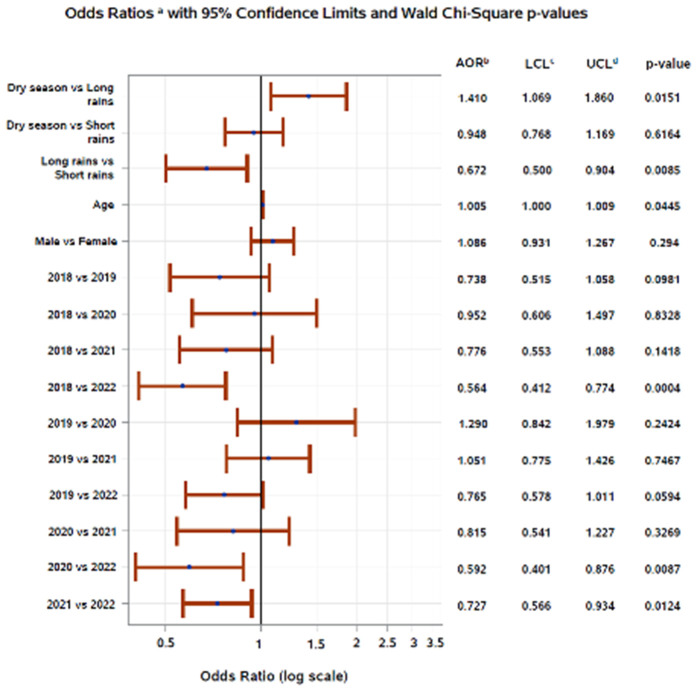
Adjusted odds of enteric pathogen detection in Addis Ababa, Ethiopia, 2018–2022. ^a^Displaying main effects, ^b^Adjusted odds ratio, ^c^Wald 95% lower confidence limit, ^d^Wald 95% upper confidence limit.

**Fig 6:**
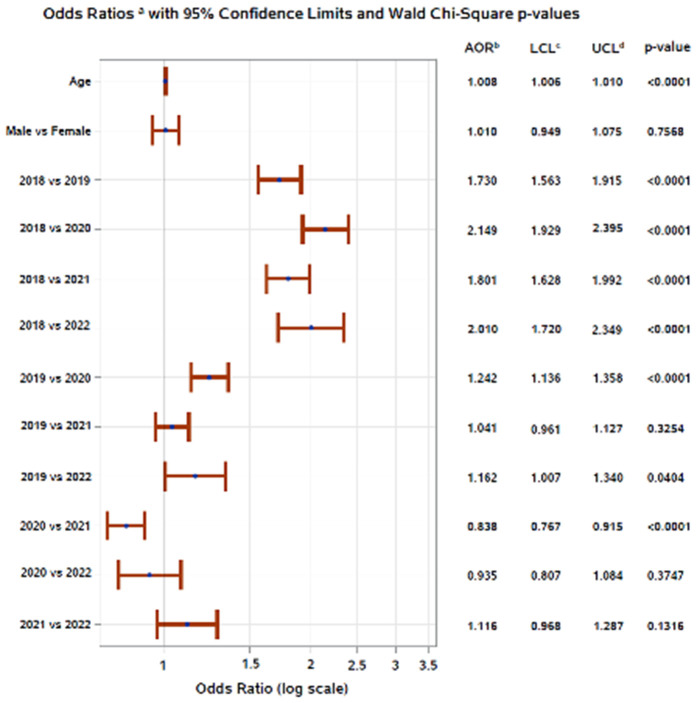
Adjusted odds of enteric pathogen detection in Gondar, Ethiopia, 2018–2022. ^a^Displaying main effects, ^b^Adjusted odds ratio, ^c^Wald 95% lower confidence limit, ^d^Wald 95% upper confidence limit.

**Fig 7:**
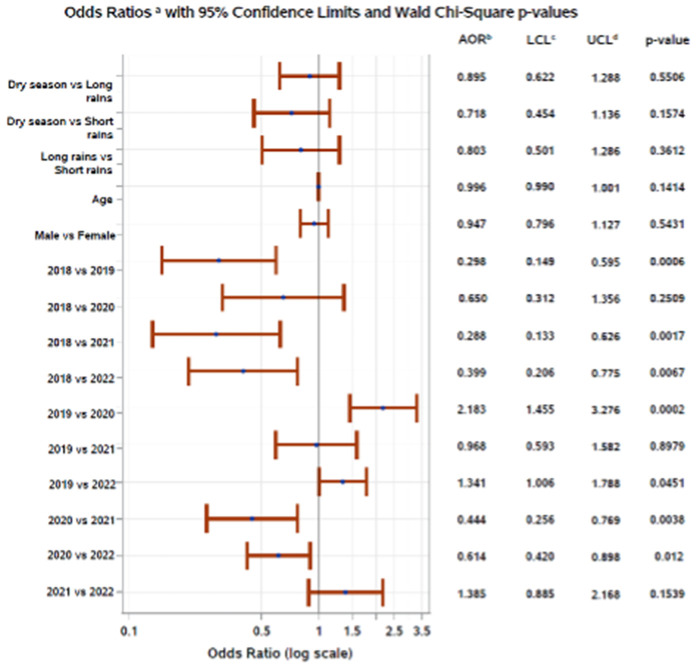
Adjusted odds of enteric pathogen detection in Harar, Ethiopia, 2018–2022. ^a^Displaying main effects, ^b^Adjusted odds ratio, ^c^Wald 95% lower confidence limit, ^d^Wald 95% upper confidence limit.

In multivariable logistic regression models, factors associated with enteric infection from bacterial or parasitic pathogens were generally consistent across study sites, with year of submission being statistically significant at all sites, when adjusting for sex. In Addis Ababa, the odds of enteric infection were higher in 2018, 2020, and 2021 compared to 2022, and samples submitted in 2020 and 2021 had higher odds of enteric infection compared to 2022 (Fig 5). In Gondar, the odds of enteric infection were higher in 2018 compared to all other years (2019, 2020, 2021, 2022); samples submitted in 2019 had higher odds of enteric infection compared to 2020 and 2022; and samples submitted in 2020 had higher odds of enteric infection compared to 2021 (Fig 6). In Harar, the odds of enteric infection were higher in 2018 compared to 2019, 2021, and 2022; the odds of enteric infection were also higher in 2019 and 2020 compared to 2022; and the odds of enteric infection were higher in 2020 compared to 2021 (Fig 7). Additionally, patient age was associated with enteric infection in Addis Ababa and Gondar, with the odds of infection increasing slightly with increasing age. Season met model inclusion criteria in Addis Ababa and Harar, but was only statistically significant in Addis Ababa, where the odds of enteric infection were higher in the dry season and short rains season compared to the long rains season. Crude and adjusted odds ratios, confidence intervals, and p-values are presented in the S4 File. Alternative models classifying age as a categorical variable can be found in the S5 File.

## Discussion

The overall prevalence of enteric infection in this study, regardless of site, was substantially lower than other reported estimates for comparable regions in Ethiopia, however, previous studies have primarily focused on schoolchildren and rural residents [6,41–43]. This might be due to limitations in laboratory testing capacity or low community healthcare-seeking behaviors related to diarrheal disease as knowledge, attitudes, and practices surrounding diarrheal disease and enteric infection are thought to differ by community [34]. Additionally, many studies reporting the prevalence of enteric infection in Ethiopia utilize active sampling approaches and often test asymptomatic individuals, while this study relied on patients seeking healthcare and submitting samples to hospital clinical laboratories. However, given that the study population was individuals with suspected gastroenteropathy who submitted a stool sample, our findings may overestimate the prevalence of enteric pathogens in the target population.

Stool sample submissions varied significantly over time at all three study sites. In Addis Ababa, submissions were generally consistent throughout the study period compared to Gondar and Harar where submissions varied significantly. This variation may be due to factors like seasonality, differing healthcare-seeking behaviors across sites, and inconsistent laboratory capacity and testing infrastructure across sites. At all study sites, stool sample submission decreased during 2020, possibly due to the COVID-19 pandemic. For example, a cross-sectional study of tertiary care clinics in Addis Ababa found that patients who feared contracting COVID-19 were 19 times more likely to miss follow-up healthcare appointments compared to patients who were not afraid of contracting COVID-19 at healthcare appointments [44]. Additionally, the same study also found that older patients and patients with limited access to transportation were more likely to miss follow-up appointments compared to younger patients and patients with access to transportation, respectively. This study suggests patients may have been less inclined to seek healthcare and submit stool samples during the COVID-19 pandemic.

The prevalence estimates for parasitic infection reported in this study are also lower than estimates from comparable regions of Ethiopia. Specifically, Addis Ababa reported a substantially lower prevalence of most enteric pathogens compared to other sites. This might be due to the predominantly urban population, improved water infrastructure, or the presence of multiple other hospitals in the area that patients may have visited for symptomatic enteric infection. Additionally, seasonal weather conditions vary geographically within Ethiopia, which could result in differences in enteric infection by study site [45]. For example, a 10-year cross-sectional study of symptomatic patients visiting a university health clinic in central Ethiopia estimated the prevalence of parasitic infection to be 47.9%, while a study of primary students in northwest Ethiopia estimated the prevalence of parasitic infection to be 65.5% [41,42]. Similarly, a study of asymptomatic food handlers in northwest Ethiopia estimated the overall prevalence of parasitic infection to be 41.1% with *Entamoeba histolytica* comprising 12.76% and *Giardia lamblia* 7.00% [18]. Our estimates are likely lower than previous studies due to limitations in routine diagnostic procedures, especially when compared to studies prospectively recruiting patients to test for parasitic pathogens. Largely, previous studies investigating enteric infection and diarrheal disease in Ethiopia have focused on asymptomatic adults or symptomatic children. However, a previous study of symptomatic patients in southern Ethiopia reported higher prevalence estimates for parasitic infection [41]. Notably, these cross-sectional studies have actively sought out individuals in the community and health clinics and conducted stool testing for enteric pathogens rather than, as in our study, utilizing data from individuals with illnesses who sought healthcare. STH estimates from this study were lower than those observed in other studies from comparable regions of Ethiopia. For example, a study of school children in South Ethiopia estimated the prevalence of STH to be 54% [46], while another study of school children near Addis Ababa (i.e., southwest Ethiopia) reported the prevalence of STH to be 45.6% [47]. Parasitic infection from *Strongyloides stercoralis*, a less frequently studied soil-transmitted helminth, can pose a significant threat to human health as the infection will not resolve without treatment and contributes to morbidity and mortality in many tropical and subtropical countries [48]. A study of school children in rural Northwest Ethiopia estimated the prevalence of *Strongyloides stercoralis* to be 3.5% with conventional methods and 13.4% with PCR, suggesting that prevalence estimates using conventional methods might be underestimated [49]. Our prevalence estimates were likely lower than previous studies in the region due to limitations in detection methods, however, existing estimates of *Strongyloides stercoralis* vary greatly.

The overall prevalence estimates of bacterial infection from *Salmonella* and *Shigella* varied by study site. Estimates in Addis Ababa were lower than expected, and estimates in Gondar were similar to previously reported estimates for comparable regions. For example, our study estimated the prevalence of *Shigella* and *Salmonella* to be 7.59% and 0%, respectively, in Gondar. Studies from the same region (i.e., northwest Ethiopia) reported prevalence rates of 4.0% for *Shigella* and 3.1% for *Salmonella* in under-five children [17]; 1.6% for *Salmonella* in seemingly healthy food handlers [18]; and 10.1% for *Shigella* and 1.2% for *Salmonella* in food handlers [19]. A study of patients with diarrhea in Addis Ababa, Ethiopia, estimated the prevalence of *Shigella* and *Salmonella* to be 5.6% and 8.4%, respectively [50], while a study of hospital food handlers in Addis Ababa estimated the prevalence of *Salmonella* to be 3.8% [20]. These estimates are higher than our estimates, but these studies conducted active surveillance for bacterial pathogens, which could explain their higher reported estimates. Additionally, the Global Pediatric Diarrhea Surveillance study, which includes a site in Ethiopia, also estimates the prevalence of *Shigella* to be higher than our study [51]. However, given that our study population was individuals submitting stool samples, it is likely that the prevalence of enteric infection is overestimated in our population. Our estimates may be lower due to differences in hospital laboratory practices and diagnostic procedures, such as some pathogens like *Campylobacter* spp. not routinely tested for in samples. Our estimates are also likely lower than expected due to limitations in diagnostic testing and laboratory capacity at the study sites, which may influence physician ordering practices around stool sample testing. For example, a study of diagnostic procedures for stool sample testing at South African public hospitals found that over 75% of doctors used personal judgment rather than clinical or diagnostic protocols [15]. The same study also found that bacterial pathogen yield was nearly 38% lower when traditional diagnostic methods were used to test stool samples rather than molecular methods. This may be due to the low sensitivity and specificity of traditional diagnostic methods.

Patient age, season, and year were significantly associated with enteric infection in this study, although the direction of association differed from previous studies. The odds of enteric infection increased with age in Addis Ababa and Gondar. This is in line with one previous study and contradicts another. For example, a study of under-five children in Gondar identified increasing age as a risk factor for parasitic infection [52]; while a study of primary school children in Gondar identified younger school children (grades 1 – 3) to have a higher risk of enteric infection compared to older school children (grades 4 – 8) [53]. Notably, the same study found that age was not significantly associated with parasitic infection despite the grade-level association. A study of STH infection in school children southwest of Addis Ababa identified seasonality as a significant risk factor for infection with prevalence and intensity of STH increasing in the dry season [54]. This corroborates our finding of increased enteric infection odds during the dry season in Addis Ababa. Increased risk of enteric infection during the dry season may be due to wastewater reuse or reliance on other unimproved water sources during periods of drought [55,56]. Alternatively, the increased risk of enteric infection observed during the dry season might be mediated via a relationship between increased temperature and enteric infection; studies have found that increasing temperatures are associated with increases in enteric infection [57,58]. The association between year and prevalence of enteric infection across study sites may be due to changes in hospital laboratory testing capacity and diagnostic procedures or events like the COVID-19 pandemic that influenced healthcare-seeking behavior during the study period [44]. No studies have assessed stool sample submission rates over time in Ethiopia; this information could potentially explain associations between enteric infection and years of submission.

Multiple parasitic codetections and one bacterial codetection were identified in this study. Pathogens frequently co-detected included *Entamoeba histolytica*, *Giardia lamblia*, and STH such as *Ascaris lumbricoides* or *Strongyloides stercoralis*. Notably, only one bacterial sample was classified as a codetection, possibly due to the small sample size or diagnostic methods. Additionally, diagnostic limitations such as testing for a select few bacterial pathogens may have allowed for gaps in codetection identification, as testing for many bacterial pathogens was not routinely conducted.

The data collected and analyzed for this work contributes to establishing a baseline for parasitic and bacterial prevalence in Ethiopian hospitals, relying on laboratory results. However, multiple limitations should be considered when interpreting our results. First, the consistency and detail of clinical laboratory records varied by study site, making it difficult to accurately compare trends across study sites as collection, testing, and reporting protocols vary by hospital. Second, there may be differences between and within study sites in how samples were collected and analyzed, which could impact the specificity of estimates; this may be due to deviations from protocols or changes in protocols. Third, detailed information on pathogen testing was not consistently available at each site and changes over time were not documented. For example, it was unclear in some cases if samples did not test positive because they were not tested for the pathogen. Fourth, data were limited to what was recorded in the logbook at the time of stool sample submission, limiting our ability to investigate other relevant factors that could influence exposure and outcomes, such as clinical symptoms, food consumption, contact with livestock, water sources, or food preparation practices. Fifth, laboratory results were collected on paper forms at the time of sample testing, introducing the possibility of some errors despite the quality checks. Sixth, these estimates are not generalizable to the entire country as the study was limited to three study sites. Finally, since the study period was short (i.e., five years) and included the COVID-19 pandemic, these results may not be representative of current trends in Ethiopia.

## Conclusion

This study demonstrates the variation in the prevalence of enteric pathogens by study site, likely due to testing protocols, healthcare-seeking behavior, and infrastructure. Since prevalence estimates differed across study sites and testing was not conducted for many enteric pathogens associated with diarrhea, country-wide surveillance is needed to better estimate the impact of diarrheal disease-associated infection in Ethiopia. Additionally, since estimates were lower than in previous studies, efforts to assess laboratory diagnostic methods are necessary to ensure effective testing across study sites. Since geographical, temporal, and seasonal patterns in enteric infection exist in Ethiopia, additional research to understand the scope of enteric infection is necessary to adequately allocate resources towards robust diagnostic procedures and increased laboratory capacity for stool testing. Robust testing of stool samples is necessary to better diagnose enteric infection and manage diarrheal disease. Efforts to mitigate enteric infection can benefit from utilizing seasonal and geographic trends in infection to anticipate areas in need of additional laboratory resources.

## Supporting information

S1 File Codebook. (XLSX)

S2 File Average monthly prevalence of enteric infection from all tested pathogens by study site. (DOCX)

S3 File Parasitic and bacterial codetections in Addis Ababa, Gondar, and Harar, Ethiopia. (DOCX)

S4 File Univariate and multivariable logistic regression analyses of factors associated with enteric infection in Ethiopia, 2018–2022. (DOCX)

S5 File Univariate and multivariable logistic regression analyses of factors associated with enteric infection in Ethiopia, 2018–2022 (alternative age classification). (DOCX)
