# Supplementary material for: Prevalence and factors associated with laboratory-confirmed cases of select enteric infections in three Ethiopian communities, 2018–2022
Source: PLOS Glob Public Health. 2025 Aug 11;5(8):e0005021. doi: 10.1371/journal.pgph.0005021 (PMC12338818; doi:10.1371/journal.pgph.0005021)
Supplement: S2 File — (DOCX) [file pgph.0005021.s002.docx]

**S2 File. Average monthly prevalence of enteric infection from all tested pathogens by study site**

| **Study Site** | **Month** | **Prevalence^a^** | **LCL^b^** | **UCL^c^** |
| --- | --- | --- | --- | --- |
| Addis Ababa | January | 4.55 | 1.09 | 8.02 |
| Gondar | January | 18.58 | -10.17 | 47.34 |
| Harar | January | 12.65 | -3.70 | 29.01 |
| Addis Ababa | February | 6.24 | 3.32 | 9.16 |
| Gondar | February | 12.67 | 2.23 | 23.10 |
| Harar | February | 15.26 | 1.80 | 28.72 |
| Addis Ababa | March | 5.31 | 3.28 | 7.33 |
| Gondar | March | 13.83 | 3.69 | 23.98 |
| Harar | March | 8.20 | 0.76 | 15.65 |
| Addis Ababa | April | 3.56 | 0.20 | 6.93 |
| Gondar | April | 13.78 | 4.18 | 23.98 |
| Harar | April | 21.42 | 3.48 | 39.36 |
| Addis Ababa | May | 4.71 | 1.21 | 8.21 |
| Gondar | May | 9.95 | -1.78 | 21.69 |
| Harar | May | 22.05 | -6.78 | 50.89 |
| Addis Ababa | June | 3.88 | 1.49 | 6.28 |
| Gondar | June | 12.07 | 0.11 | 24.03 |
| Harar | June | 17.15 | 0.92 | 33.39 |
| Addis Ababa | July | 3.75 | 1.39 | 6.12 |
| Gondar | July | 14.08 | 3.60 | 24.56 |
| Harar | July | 13.65 | 3.38 | 23.93 |
| Addis Ababa | August | 2.04 | -0.43 | 4.51 |
| Gondar | August | 17.28 | 10.57 | 24.00 |
| Harar | August | 16.12 | -9.28 | 41.52 |
| Addis Ababa | September | 4.03 | 0.99 | 7.08 |
| Gondar | September | 19.08 | 10.58 | 27.59 |
| Harar | September | 19.11 | 2.15 | 36.06 |
| Addis Ababa | October | 2.47 | 0.59 | 4.34 |
| Gondar | October | 18.54 | 11.81 | 25.28 |
| Harar | October | 10.30 | -8.08 | 28.68 |
| Addis Ababa | November | 3.28 | -1.34 | 7.90 |
| Gondar | November | 17.11 | 9.10 | 25.11 |
| Harar | November | 19.80 | -12.40 | 52.00 |
| Addis Ababa | December | 3.24 | 0.73 | 5.75 |
| Gondar | December | 19.94 | 8.51 | 31.37 |
| Harar | December | 10.96 | 5.94 | 15.98 |

^a^ Months with no submissions excluded from prevalence estimates

^b^ Wald 95% Lower Confidence Limit

^c^ Wald 95% Upper Confidence Limit
