# Supplementary material for: Prevalence and factors associated with laboratory-confirmed cases of select enteric infections in three Ethiopian communities, 2018–2022
Source: PLOS Glob Public Health. 2025 Aug 11;5(8):e0005021. doi: 10.1371/journal.pgph.0005021 (PMC12338818; doi:10.1371/journal.pgph.0005021)
Supplement: S3 File — (DOCX) [file pgph.0005021.s003.docx]

**S3 File. Parasitic and bacterial codetections in Addis Ababa, Gondar, and Harar, Ethiopia, 2018 – 2022**


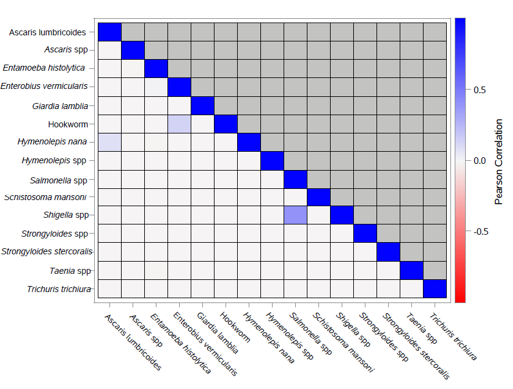


**Study site: Addis Ababa**


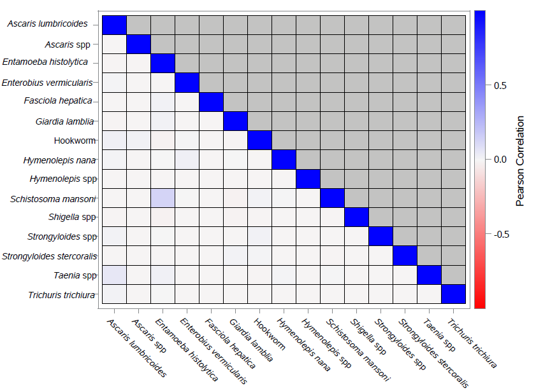


**Study site: Gondar**

**
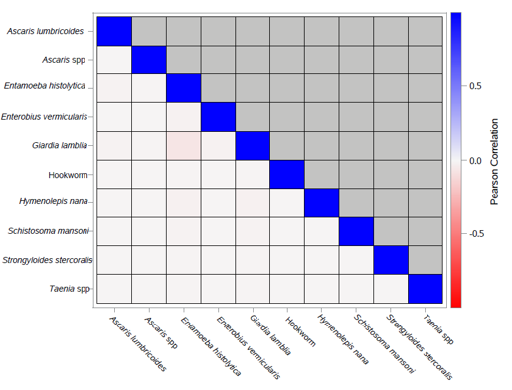
**

**Study site: Harar**
