# Supplementary material for: Prevalence and factors associated with laboratory-confirmed cases of select enteric infections in three Ethiopian communities, 2018–2022
Source: PLOS Glob Public Health. 2025 Aug 11;5(8):e0005021. doi: 10.1371/journal.pgph.0005021 (PMC12338818; doi:10.1371/journal.pgph.0005021)
Supplement: S4 File — (DOCX) [file pgph.0005021.s004.docx]

**S4 File. Univariate and multivariable logistic regression analyses of factors associated with enteric infection in Ethiopia, 2018 - 2022**

| **Addis Ababa** | | | | | |
| --- | --- | --- | --- | --- | --- |
| **Factor** | | **COR^a^ (95% CI^b^)** | **p-value** | **AOR^c^ (95% CI)** | **p-value** |
| Age | | 1.007 (1.003, 1.010) | 0.0002 | 1.005 (1.000, 1.009) | 0.0445 |
| Sex | Male vs Female | 1.076 (0.927, 1.248) | 0.3355 | 1.086 (0.931, 1.267) | 0.2940 |
| Season | Dry vs Long rains | 1.126 (0.937, 1.353) | 0.2046 | 1.410 (1.069, 1.860) | 0.0151 |
|  | Dry vs Short rains | 0.970 (0.813, 1.157) | 0.7353 | 0.948 (0.768, 1.169) | 0.6164 |
|  | Long rains vs Short rains | 0.861 (0.709, 1.047) | 0.1344 | 0.672 (0.500, 0.904) | 0.0085 |
| Year^d^ | 2018 vs 2019 | 0.963 (0.748, 1.240) | 0.7689 | 0.738 (0.515, 1.058) | 0.0981 |
|  | 2018 vs 2020 | 1.087 (0.786, 1.503) | 0.6144 | 0.952 (0.606, 1.497) | 0.8328 |
|  | 2018 vs 2021 | 0.828 (0.656, 1.044) | 0.1101 | 0.776 (0.553, 1.088) | 0.1418 |
|  | 2018 vs 2022 | 0.708 (0.559, 0.895) | 0.0039 | 0.564 (0.412, 0.774) | 0.0004 |
|  | 2019 vs 2020 | 1.129 (0.824, 1.546) | 0.4497 | 1.290 (0.842, 1.979) | 0.2424 |
|  | 2019 vs 2021 | 0.860 (0.691, 1.069) | 0.1741 | 1.051 (0.775, 1.426) | 0.7467 |
|  | 2019 vs 2022 | 0.735 (0.589, 0.917) | 0.0064 | 0.765 (0.578, 1.011) | 0.0594 |
|  | 2020 vs 2021 | 0.761 (0.565, 1.026) | 0.0730 | 0.815 (0.541, 1.227) | 0.3269 |
|  | 2020 vs 2022 | 0.651 (0.482, 0.879) | 0.0051 | 0.592 (0.401, 0.876) | 0.0087 |
|  | 2021 vs 2022 | 0.855 (0.702, 1.041) | 0.1190 | 0.727 (0.566, 0.934) | 0.0124 |
| Age*Year |  |  |  |  | 0.0376 |
| Season*Year |  |  |  |  | 0.0062 |
| Season*Sex |  |  |  |  | 0.0027 |

| **Gondar** | | | | | |
| --- | --- | --- | --- | --- | --- |
| **Factor** | | **COR (95% CI)** | **p-value** | **AOR (95% CI)** | **p-value** |
| Age | | 1.008 (1.006, 1.009) | <0.0001 | 1.008 (1.006, 1.010) | <0.0001 |
| Sex | Male vs Female | 1.019 (0.958, 1.083) | 0.5571 | 1.010 (0.9490, 1.075) | 0.7568 |
| Season | Dry vs Long rains | 0.958 (0.895, 1.026) | 0.2193 |  |  |
|  | Dry vs Short rains | 1.012 (0.931, 1.100) | 0.7812 |  |  |
|  | Long rains vs Short rains | 1.056 (0.971, 1.149) | 0.2060 |  |  |
| Year | 2018 vs 2019 | 1.699 (1.535, 1.879) | <0.0001 | 1.730 (1.563, 1.915) | <0.0001 |
|  | 2018 vs 2020 | 2.130 (1.912, 2.371) | <0.0001 | 2.149 (1.929, 2.395) | <0.0001 |
|  | 2018 vs 2021 | 1.742 (1.578, 1.924) | <0.0001 | 1.801 (1.628, 1.992) | <0.0001 |
|  | 2018 vs 2022 | 1.936 (1.664, 2.252) | <0.0001 | 2.010 (1.720, 2.349) | <0.0001 |
|  | 2019 vs 2020 | 1.254 (1.147, 1.370) | <0.0001 | 1.242 (1.136, 1.358) | <0.0001 |
|  | 2019 vs 2021 | 1.026 (0.948, 1.110) | 0.5239 | 1.041 (0.961, 1.127) | 0.3254 |
|  | 2019 vs 2022 | 1.140 (0.992, 1.309) | 0.0647 | 1.162 (1.007, 1.340) | 0.0404 |
|  | 2020 vs 2021 | 0.818 (0.750, 0.892) | <0.0001 | 0.838 (0.767, 0.915) | <0.0001 |
|  | 2020 vs 2022 | 0.909 (0.787, 1.049) | 0.1926 | 0.935 (0.807, 1.084) | 0.3747 |
|  | 2021 vs 2022 | 1.111 (0.968, 1.275) | 0.1338 | 1.116 (0.968, 1.287) | 0.1316 |
| Age*Year |  |  |  |  | 0.0258 |

| **Harar** | | | | | |
| --- | --- | --- | --- | --- | --- |
| **Factor** | | **COR (95% CI)** | **p-value** | **AOR (95% CI)** | **p-value** |
| Age | | 0.994 (0.989, 0.999) | 0.0216 | 0.996 (0.990, 1.001) | 0.1414 |
| Sex | Male vs Female | 0.925 (0.780, 1.097) | 0.3718 | 0.947 (0.796, 1.127) | 0.5431 |
| Season | Dry vs Long rains | 0.654 (0.530, 0.806) | <0.0001 | 0.895 (0.622, 1.288) | 0.5506 |
|  | Dry vs Short rains | 0.485 (0.392, 0.600) | <0.0001 | 0.718 (0.454, 1.136) | 0.1574 |
|  | Long rains vs Short rains | 0.742 (0.602, 0.913) | 0.0048 | 0.803 (0.501, 1.286) | 0.3612 |
| Year | 2018 vs 2019 | 0.243 (0.125, 0.473) | <0.0001 | 0.298 (0.149, 0.595) | 0.0006 |
|  | 2018 vs 2020 | 0.521 (0.265, 1.025) | 0.0588 | 0.650 (0.312, 1.356) | 0.2509 |
|  | 2018 vs 2021 | 0.402 (0.204, 0.790) | 0.0082 | 0.288 (0.133, 0.626) | 0.0017 |
|  | 2018 vs 2022 | 0.396 (0.205, 0.762) | 0.0055 | 0.399 (0.206, 0.775) | 0.0067 |
|  | 2019 vs 2020 | 2.144 (1.629, 2.822) | <0.0001 | 2.183 (1.455, 3.276) | 0.0002 |
|  | 2019 vs 2021 | 1.653 (1.255, 2.176) | 0.0003 | 0.968 (0.593, 1.582) | 0.8979 |
|  | 2019 vs 2022 | 1.627 (1.305. 2.028) | 0.0001 | 1.341 (1.006, 1.788) | 0.0451 |
|  | 2020 vs 2021 | 0.771 (0.572, 1.039) | 0.0880 | 0.444 (0.256, 0.769) | 0.0038 |
|  | 2020 vs 2022 | 0.759 (0.591, 0.974) | 0.0302 | 0.614 (0.420, 0.898) | 0.0120 |
|  | 2021 vs 2022 | 0.985 (0.767, 1.264) | 0.9030 | 1.385 (0.885, 2.168) | 0.1539 |
| Season*Year |  |  |  |  | 0.0190 |

^a^ Crude Odds Ratio

^b^ 95% Wald Confidence Interval

^c^ Adjusted Odds Ratio
