# Supplementary material for: Prevalence and factors associated with laboratory-confirmed cases of select enteric infections in three Ethiopian communities, 2018–2022
Source: PLOS Glob Public Health. 2025 Aug 11;5(8):e0005021. doi: 10.1371/journal.pgph.0005021 (PMC12338818; doi:10.1371/journal.pgph.0005021)
Supplement: S5 File — (DOCX) [file pgph.0005021.s005.docx]

**S5 File. Univariate and multivariable logistic regression analyses of factors associated with enteric infection in Ethiopia, 2018 – 2022 (alternative age classification)**

| **Addis Ababa** | | | | | |
| --- | --- | --- | --- | --- | --- |
| **Factor** | | **COR^a^ (95% CI^b^)** | **p-value** | **AOR^c^ (95% CI)** | **p-value** |
| Age | 0-23 months vs 24-59 months | 0.444 (0.309, 0.637) | <0.0001 | 0.452 (0.315, 0.650) | <0.0001 |
|  | 0-23 months vs 5-9 years | 0.354 (0.246, 0.509) | <0.0001 | 0.367 (0.255, 0.528) | <0.0001 |
|  | 0-23 months vs 10-14 years | 0.354 (0.238, 0.526) | <0.0001 | 0.367 (0.247, 0.546) | <0.0001 |
|  | 0-23 months vs 15-19 years | 0.351 (0.224, 0.550) | <0.0001 | 0.366 (0.232, 0.576) | <0.0001 |
|  | 0-23 months vs 20-29 years | 0.386 (0.276, 0.540) | <0.0001 | 0.399 (0.284, 0.562) | <0.0001 |
|  | 0-23 months vs 30-39 years | 0.387 (0.271, 0.553) | <0.0001 | 0.402 (0.279, 0.578) | <0.0001 |
|  | 0-23 months vs 40-64 years | 0.436 (0.309, 0.617) | <0.0001 | 0.469 (0.329, 0.669) | <0.0001 |
|  | 0-23 months vs 65+ years | 0.285 (0.190, 0.425) | <0.0001 | 0.310 (0.206, 0.468) | <0.0001 |
|  | 24-59 months vs 5-9 years | 0.797 (0.588, 1.081) | 0.1441 | 0.811 (0.598, 1.101) | 0.1791 |
|  | 24-59 months vs 10-14 years | 0.798 (0.567, 1.124) | 0.1966 | 0.812 (0.576, 1.144) | 0.2331 |
|  | 24-59 months vs 15-19 years | 0.792 (0.529, 1.184) | 0.2551 | 0.809 (0.538, 1.217) | 0.3088 |
|  | 24-59 months vs 20-29 years | 0.871 (0.665, 1.141) | 0.3152 | 0.883 (0.670, 1.164) | 0.3782 |
|  | 24-59 months vs 30-39 years | 0.873 (0.649, 1.174) | 0.3680 | 0.888 (0.655, 1.204) | 0.4459 |
|  | 24-59 months vs 40-64 years | 0.983 (0.741, 1.305) | 0.9069 | 1.036 (0.772, 1.390) | 0.8125 |
|  | 24-59 months vs 65+ years | 0.641 (0.452, 0.910) | 0.0127 | 0.685 (0.479, 0.981) | 0.0391 |
|  | 5-9 years vs 10-14 years | 1.001 (0.710, 1.412) | 0.9946 | 1.000 (0.709, 1.412) | 0.9981 |
|  | 5-9 years vs 15-19 years | 0.993 (0.663, 1.487) | 0.9725 | 0.997 (0.662, 1.501) | 0.9879 |
|  | 5-9 years vs 20-29 years | 1.092 (0.832, 1.434) | 0.5246 | 1.088 (0.824, 1.438) | 0.5509 |
|  | 5-9 years vs 30-39 years | 1.095 (0.812, 1.475) | 0.5522 | 1.095 (0.806, 1.487) | 0.5617 |
|  | 5-9 years vs 40-64 years | 1.233 (0.928, 1.640) | 0.1491 | 1.277 (0.950, 1.716) | 0.1047 |
|  | 5-9 years vs 65+ years | 0.804 (0.566, 1.143) | 0.2243 | 0.845 (0.589, 1.211) | 0.3584 |
|  | 10-14 years vs 15-19 years | 0.992 (0.643, 1.530) | 0.9701 | 0.996 (0.642, 1.546) | 0.9872 |
|  | 10-14 years vs 20-29 years | 1.091 (0.797, 1.493) | 0.5866 | 1.088 (0.790, 1.499) | 0.6060 |
|  | 10-14 years vs 30-39 years | 1.093 (0.781, 1.532) | 0.6036 | 1.094 (0.775, 1.545) | 0.6079 |
|  | 10-14 years vs 40-64 years | 1.232 (0.890, 1.706) | 0.2089 | 1.277 (0.913, 1.785) | 0.1537 |
|  | 10-14 years vs 65+ years | 0.803 (0.547, 1.180) | 0.2647 | 0.844 (0.570, 1.252) | 0.3999 |
|  | 15-19 years vs 20-29 years | 1.100 (0.753, 1.607) | 0.6217 | 1.092 (0.747, 1.596) | 0.6503 |
|  | 15-19 years vs 30-39 years | 1.102 (0.740, 1.642) | 0.6310 | 1.098 (0.737, 1.637) | 0.6452 |
|  | 15-19 years vs 40-64 years | 1.242 (0.842, 1.831) | 0.2736 | 1.281 (0.867, 1.892) | 0.2131 |
|  | 15-19 years vs 65+ years | 0.810 (0.522, 1.257) | 0.3473 | 0.847 (0.545, 1.318) | 0.4622 |
|  | 20-29 years vs 30-39 years | 1.002 (0.770, 1.304) | 0.9870 | 1.006 (0.772, 1.310) | 0.9649 |
|  | 20-29 years vs 40-64 years | 1.129 (0.881, 1.447) | 0.3374 | 1.173 (0.913, 1.507) | 0.2107 |
|  | 20-29 years vs 65+ years | 0.736 (0.534, 1.016) | 0.0625 | 0.776 (0.561, 1.074) | 0.1261 |
|  | 30-39 years vs 40-64 years | 1.127 (0.854, 1.486) | 0.3983 | 1.166 (0.884, 1.540) | 0.2774 |
|  | 30-39 years vs 65+ years | 0.735 (0.521, 1.037) | 0.0795 | 0.772 (0.546, 1.091) | 0.1422 |
|  | 40-64 years vs 65+ years | 0.652 (0.468, 0.910) | 0.0119 | 0.661 (0.474, 0.924) | 0.0154 |
| Sex | Male vs Female | 1.076 (0.927, 1.248) | 0.3355 | 1.103 (0.945, 1.287) | 0.2151 |
| Season | Dry vs Long rains | 1.126 (0.937, 1.353) | 0.2046 | 1.408 (1.067, 1.858) | 0.0157 |
|  | Dry vs Short rains | 0.970 (0.813, 1.157) | 0.7353 | 0.938 (0.760, 1.158) | 0.5535 |
|  | Long rains vs Short rains | 0.861 (0.709, 1.047) | 0.1344 | 0.666 (0.495, 0.896) | 0.0073 |
| Year | 2018 vs 2019 | 0.963 (0.748, 1.240) | 0.7689 | 0.721 (0.520, 0.999) | 0.0493 |
|  | 2018 vs 2020 | 1.087 (0.786, 1.503) | 0.6144 | 0.975 (0.625, 1.521) | 0.9106 |
|  | 2018 vs 2021 | 0.828 (0.656, 1.044) | 0.1101 | 0.774 (0.553, 1.081) | 0.1328 |
|  | 2018 vs 2022 | 0.708 (0.559, 0.895) | 0.0039 | 0.608 (0.446, 0.829) | 0.0016 |
|  | 2019 vs 2020 | 1.129 (0.824, 1.546) | 0.4497 | 1.353 (0.905, 2.021) | 0.1404 |
|  | 2019 vs 2021 | 0.860 (0.691, 1.069) | 0.1741 | 1.073 (0.823, 1.400) | 0.6020 |
|  | 2019 vs 2022 | 0.735 (0.589, 0.917) | 0.0064 | 0.844 (0.663, 1.074) | 0.1674 |
|  | 2020 vs 2021 | 0.761 (0.565, 1.026) | 0.0730 | 0.794 (0.529, 1.189) | 0.2628 |
|  | 2020 vs 2022 | 0.651 (0.482, 0.879) | 0.0051 | 0.624 (0.426, 0.914) | 0.0155 |
|  | 2021 vs 2022 | 0.855 (0.702, 1.041) | 0.1190 | 0.786 (0.614, 1.007) | 0.0568 |
| Season*Year |  |  |  |  | 0.0067 |
| Season*Sex |  |  |  |  | 0.0032 |

| **Gondar** | | | | | |
| --- | --- | --- | --- | --- | --- |
| **Factor** | | **COR (95% CI)** | **p-value** | **AOR (95% CI)** | **p-value** |
| Age | 0-23 months vs 24-59 months | 0.373 (0.290, 0.480) | <0.0001 | 0.364 (0.281, 0.471) | <0.0001 |
|  | 0-23 months vs 5-9 years | 0.285 (0.221, 0.368) | <0.0001 | 0.281 (0.217, 0.365) | <0.0001 |
|  | 0-23 months vs 10-14 years | 0.272 (0.209, 0.354) | <0.0001 | 0.277 (0.211, 0.364) | <0.0001 |
|  | 0-23 months vs 15-19 years | 0.230 (0.180, 0.293) | <0.0001 | 0.218 (0.170, 0.280) | <0.0001 |
|  | 0-23 months vs 20-29 years | 0.251(0.202, 0.312) | <0.0001 | 0.233 (0.187, 0.292) | <0.0001 |
|  | 0-23 months vs 30-39 years | 0.264 (0.211, 0.330) | <0.0001 | 0.250 (0.198, 0.315) | <0.0001 |
|  | 0-23 months vs 40-64 years | 0.249 (0.199, 0.311) | <0.0001 | 0.231 (0.184, 0.291) | <0.0001 |
|  | 0-23 months vs 65+ years | 0.221(0.172, 0.285) | <0.0001 | 0.213 (0.164, 0.276) | <0.0001 |
|  | 24-59 months vs 5-9 years | 0.766 (0.628, 0.933) | 0.0082 | 0.773 (0.632, 0.946) | 0.0124 |
|  | 24-59 months vs 10-14 years | 0.730 (0.591, 0.901) | 0.0034 | 0.761 (0.613, 0.944) | 0.0131 |
|  | 24-59 months vs 15-19 years | 0.616 (0.514, 0.740) | <0.0001 | 0.599 (0.498, 0.721) | <0.0001 |
|  | 24-59 months vs 20-29 years | 0.674 (0.582, 0.781) | <0.0001 | 0.642 (0.552, 0.746) | <0.0001 |
|  | 24-59 months vs 30-39 years | 0.707 (0.605, 0.827) | <0.0001 | 0.687 (0.585, 0.807) | <0.0001 |
|  | 24-59 months vs 40-64 years | 0.667 (0.571, 0.780) | <0.0001 | 0.635 (0.542, 0.744) | <0.0001 |
|  | 24-59 months vs 65+ years | 0.594 (0.488, 0.723) | <0.0001 | 0.585 (0.478, 0.715) | <0.0001 |
|  | 5-9 years vs 10-14 years | 0.953 (0.770, 1.179) | 0.6567 | 0.983 (0.790, 1.224) | 0.8813 |
|  | 5-9 years vs 15-19 years | 0.805 (0.670, 0.968) | 0.0211 | 0.775 (0.642, 0.935) | 0.0079 |
|  | 5-9 years vs 20-29 years | 0.881 (0.758, 1.023) | 0.0973 | 0.830 (0.711, 0.968) | 0.0176 |
|  | 5-9 years vs 30-39 years | 0.924 (0.788, 1.083) | 0.3266 | 0.889 (0.754, 1.048) | 0.1599 |
|  | 5-9 years vs 40-64 years | 0.871 (0.744, 1.021) | 0.0888 | 0.821 (0.699, 0.966) | 0.0172 |
|  | 5-9 years vs 65+ years | 0.776 (0.636, 0.946) | 0.0121 | 0.756 (0.617, 0.926) | 0.0070 |
|  | 10-14 years vs 15-19 years | 0.845 (0.693, 1.030) | 0.0957 | 0.788 (0.643, 0.966) | 0.0220 |
|  | 10-14 years vs 20-29 years | 0.924 (0.782, 1.092) | 0.3560 | 0.844 (0.710, 1.003) | 0.0542 |
|  | 10-14 years vs 30-39 years | 0.969 (0.814, 1.155) | 0.7259 | 0.904 (0.753, 1.084) | 0.2765 |
|  | 10-14 years vs 40-64 years | 0.914 (0.768, 1.089) | 0.3156 | 0.835 (0.698, 1.000) | 0.0498 |
|  | 10-14 years vs 65+ years | 0.814 (0.659, 1.006) | 0.0564 | 0.769 (0.618, 0.956) | 0.0181 |
|  | 15-19 years vs 20-29 years | 1.094 (0.962, 1.244) | 0.1691 | 1.071 (0.939, 1.221) | 0.3082 |
|  | 15-19 years vs 30-39 years | 1.147 (0.999, 1.318) | 0.0525 | 1.147 (0.994, 1.324) | 0.0607 |
|  | 15-19 years vs 40-64 years | 1.082 (0.943, 1.243) | 0.2620 | 1.060 (0.921, 1.220) | 0.4171 |
|  | 15-19 years vs 65+ years | 0.963 (0.802, 1.156) | 0.6875 | 0.975 (0.809, 1.176) | 0.7938 |
|  | 20-29 years vs 30-39 years | 1.048 (0.960, 1.145) | 0.2932 | 1.071 (0.976, 1.176) | 0.1486 |
|  | 20-29 years vs 40-64 years | 0.989 (0.906, 1.080) | 0.8077 | 0.990 (0.906, 1.082) | 0.8230 |
|  | 20-29 years vs 65+ years | 0.880 (0.759, 1.021) | 0.0916 | 0.911 (0.782, 1.061) | 0.2303 |
|  | 30-39 years vs 40-64 years | 0.944 (0.852, 1.045) | 0.2648 | 0.924 (0.831, 1.027) | 0.1429 |
|  | 30-39 years vs 65+ years | 0.840 (0.718, 0.983) | 0.0294 | 0.850 (0.723, 1.001) | 0.0508 |
|  | 40-64 years vs 65+ years | 0.890 (0.761, 1.041) | 0.1451 | 0.920 (0.784, 1.080) | 0.3094 |
| Sex | Male vs Female | 1.019 (0.958, 1.083) | 0.5571 | 1.142 (1.044, 1.250) | 0.0038 |
| Season | Dry vs Long rains | 0.958 (0.895, 1.026) | 0.2193 |  |  |
|  | Dry vs Short rains | 1.012 (0.931, 1.100) | 0.7812 |  |  |
|  | Long rains vs Short rains | 1.056 (0.971, 1.149) | 0.2060 |  |  |
| Year | 2018 vs 2019 | 1.699 (1.535, 1.879) | <0.0001 | 1.768 (1.596, 1.958) | <0.0001 |
|  | 2018 vs 2020 | 2.130 (1.912, 2.371) | <0.0001 | 2.141 (1.920, 2.387) | <0.0001 |
|  | 2018 vs 2021 | 1.742 (1.578, 1.924) | <0.0001 | 1.941 (1.753, 2.149) | <0.0001 |
|  | 2018 vs 2022 | 1.936 (1.664, 2.252) | <0.0001 | 2.166 (1.859, 2.524) | <0.0001 |
|  | 2019 vs 2020 | 1.254 (1.147, 1.370) | <0.0001 | 1.211 (1.108, 1.324) | <0.0001 |
|  | 2019 vs 2021 | 1.026 (0.948, 1.110) | 0.5239 | 1.098 (1.014, 1.188) | 0.0218 |
|  | 2019 vs 2022 | 1.140 (0.992, 1.309) | 0.0647 | 1.225 (1.066, 1.408) | 0.0043 |
|  | 2020 vs 2021 | 0.818 (0.750, 0.892) | <0.0001 | 0.906 (0.830, 0.990) | 0.0289 |
|  | 2020 vs 2022 | 0.909 (0.787, 1.049) | 0.1926 | 1.012 (0.876, 1.169) | 0.8737 |
|  | 2021 vs 2022 | 1.111 (0.968, 1.275) | 0.1338 | 1.116 (0.972, 1.281) | 0.1182 |
| Age*Sex |  |  |  |  | 0.0098 |

| **Harar** | | | | | |
| --- | --- | --- | --- | --- | --- |
| **Factor** | | **COR (95% CI)** | **p-value** | **AOR (95% CI)** | **p-value** |
| Age | 0-23 months vs 24-59 months | 0.458 (0.296, 0.711) | 0.0005 | 0.494 (0.317,0.771) | 0.0019 |
|  | 0-23 months vs 5-9 years | 0.492 (0.305, 0.792) | 0.0036 | 0.542 (0.333, 0.881) | 0.0134 |
|  | 0-23 months vs 10-14 years | 0.627 (0.375, 1.047) | 0.0744 | 0.697 (0.413, 1.177) | 0.1766 |
|  | 0-23 months vs 15-19 years | 0.699 (0.437, 1.117) | 0.1346 | 0.720 (0.431, 1.204) | 0.2107 |
|  | 0-23 months vs 20-29 years | 0.619 (0.419, 0.914) | 0.0158 | 0.648 (0.416, 1.008) | 0.0545 |
|  | 0-23 months vs 30-39 years | 0.809 (0.531, 1.235) | 0.3262 | 0.841 (0.526, 1.343) | 0.4676 |
|  | 0-23 months vs 40-64 years | 0.821 (0.542, 1.245) | 0.3538 | 0.834 (0.521, 1.333) | 0.4478 |
|  | 0-23 months vs 65+ years | 0.905 (0.474, 1.729) | 0.7625 | 0.862 (0.434, 1.712) | 0.6717 |
|  | 24-59 months vs 5-9 years | 1.072 (0.713, 1.612) | 0.7367 | 1.096 (0.724, 1.658) | 0.6662 |
|  | 24-59 months vs 10-14 years | 1.367 (0.873, 2.142) | 0.1715 | 1.409 (0.891, 2.228) | 0.1424 |
|  | 24-59 months vs 15-19 years | 1.525 (1.025, 2.269) | 0.0375 | 1.457 (0.934, 2.273) | 0.0974 |
|  | 24-59 months vs 20-29 years | 1.350 (1.000, 1.822) | 0.0500 | 1.310 (0.913, 1.879) | 0.1421 |
|  | 24-59 months vs 30-39 years | 1.765 (1.255, 2.484) | 0.0011 | 1.700 (1.148, 2.516) | 0.0081 |
|  | 24-59 months vs 40-64 years | 1.792 (1.284, 2.501) | 0.0006 | 1.686 (1.137, 2.500) | 0.0094 |
|  | 24-59 months vs 65+ years | 1.974 (1.086, 3.589) | 0.0258 | 1.743 (0.922, 3.296) | 0.0872 |
|  | 5-9 years vs 10-14 years | 1.275 (0.784, 2.074) | 0.3273 | 1.286 (0.785, 2.109) | 0.3183 |
|  | 5-9 years vs 15-19 years | 1.422 (0.916, 2.207) | 0.1166 | 1.330 (0.825, 2.145) | 0.2424 |
|  | 5-9 years vs 20-29 years | 1.259 (0.883, 1.793) | 0.2026 | 1.196 (0.801, 1.785) | 0.3815 |
|  | 5-9 years vs 30-39 years | 1.646 (1.115, 2.430) | 0.0121 | 1.552 (1.010, 2.384) | 0.0450 |
|  | 5-9 years vs 40-64 years | 1.671 (1.140, 2.450) | 0.0086 | 1.539 (1.000, 2.368) | 0.0498 |
|  | 5-9 years vs 65+ years | 1.841 (0.984, 3.445) | 0.0563 | 1.591 (0.822, 3.080) | 0.1680 |
|  | 10-14 years vs 15-19 years | 1.115 (0.691, 1.799) | 0.6549 | 1.034 (0.624, 1.713) | 0.8971 |
|  | 10-14 years vs 20-29 years | 0.987 (0.661, 1.474) | 0.9495 | 0.930 (0.604, 1.432) | 0.7412 |
|  | 10-14 years vs 30-39 years | 1.291 (0.838, 1.989) | 0.2468 | 1.206 (0.762, 1.909) | 0.4235 |
|  | 10-14 years vs 40-64 years | 1.310 (0.856, 2.006) | 0.2139 | 1.197 (0.756, 1.894) | 0.4436 |
|  | 10-14 years vs 65+ years | 1.444 (0.751, 2.777) | 0.2712 | 1.237 (0.627, 2.441) | 0.5391 |
|  | 15-19 years vs 20-29 years | 0.885 (0.629, 1.247) | 0.4852 | 0.899 (0.635, 1.274) | 0.5501 |
|  | 15-19 years vs 30-39 years | 1.158 (0.792, 1.691) | 0.4489 | 1.167 (0.794, 1.714) | 0.4319 |
|  | 15-19 years vs 40-64 years | 1.175 (0.810, 1.704) | 0.3956 | 1.157 (0.793, 1.690) | 0.4491 |
|  | 15-19 years vs 65+ years | 1.295 (0.696, 2.407) | 0.4145 | 1.197 (0.638, 2.243) | 0.5755 |
|  | 20-29 years vs 30-39 years | 1.308 (0.993, 1.722) | 0.0559 | 1.297 (0.982, 1.714) | 0.0669 |
|  | 20-29 years vs 40-64 years | 1.327 (1.018, 1.730) | 0.0364 | 1.287 (0.984, 1.684) | 0.0659 |
|  | 20-29 years vs 65+ years | 1.462 (0.833, 2.567) | 0.1855 | 1.331 (0.753, 2.352) | 0.3255 |
|  | 30-39 years vs 40-64 years | 1.015 (0.744, 1.385) | 0.9259 | 0.992 (0.724, 1.359) | 0.9599 |
|  | 30-39 years vs 65+ years | 1.118 (0.623, 2.009) | 0.7085 | 1.026 (0.567, 1.855) | 0.9332 |
|  | 40-64 years vs 65+ years | 1.102 (0.616, 1.970) | 0.7437 | 1.034 (0.575, 1.860) | 0.9111 |
| Sex | Male vs Female | 0.925 (0.780, 1.097) | 0.3718 | 0.957 (0.802, 1.141) | 0.6239 |
| Season | Dry vs Long rains | 0.654 (0.530, 0.806) | <0.0001 | 0.894 (0.620, 1.288) | 0.5469 |
|  | Dry vs Short rains | 0.485 (0.392, 0.600) | <0.0001 | 0.722 (0.456, 1.143) | 0.1649 |
|  | Long rains vs Short rains | 0.742 (0.602, 0.913) | 0.0048 | 0.808 (0.503, 1.297) | 0.3772 |
| Year | 2018 vs 2019 | 0.243 (0.125, 0.473) | <0.0001 | 0.300 (0.149, 0.605) | 0.0008 |
|  | 2018 vs 2020 | 0.521 (0.265, 1.025) | 0.0588 | 0.641 (0.305, 1.348) | 0.2413 |
|  | 2018 vs 2021 | 0.402 (0.204, 0.790) | 0.0082 | 0.284 (0.130, 0.618) | 0.0015 |
|  | 2018 vs 2022 | 0.396 (0.205, 0.762) | 0.0055 | 0.390 (0.201, 0.759) | 0.0055 |
|  | 2019 vs 2020 | 2.144 (1.629, 2.822) | <0.0001 | 2.136 (1.421, 3.210) | 0.0003 |
|  | 2019 vs 2021 | 1.653 (1.255, 2.176) | 0.0003 | 0.945 (0.565, 1.581) | 0.8292 |
|  | 2019 vs 2022 | 1.627 (1.305. 2.028) | 0.0001 | 1.300 (0.946, 1.788) | 0.1059 |
|  | 2020 vs 2021 | 0.771 (0.572, 1.039) | 0.0880 | 0.442 (0.250, 0.782) | 0.005 |
|  | 2020 vs 2022 | 0.759 (0.591, 0.974) | 0.0302 | 0.609 (0.408, 0.909) | 0.0152 |
|  | 2021 vs 2022 | 0.985 (0.767, 1.264) | 0.9030 | 1.376 (0.878, 2.156) | 0.1635 |
| Season*Year |  |  |  |  | 0.0208 |

^a^ Crude Odds Ratio

^b^ 95% Wald Confidence Interval

^c^ Adjusted Odds Ratio
